# Tip-multi-breaking in Capillary Microfluidic Devices

**DOI:** 10.1038/srep11102

**Published:** 2015-06-16

**Authors:** Pingan Zhu, Tiantian Kong, Zhanxiao Kang, Xiaowei Tian, Liqiu Wang

**Affiliations:** 1 Department of Mechanical Engineering, the University of Hong Kong, Hong Kong; 2 HKU-Zhejiang Institute of Research and Innovation (HKU-ZIRI), 311300, Hangzhou, Zhejiang, China

## Abstract

We report tip-multi-breaking (TMB) mode of droplet breakup in capillary microfluidic devices. This new mode appears in a region embraced by *Ca*_i_ = 0 and lg(*Ca*_*i*_) = − 8.371(*Ca*_0_) −7.36 with *Ca*_0_ varying from 0.35 to 0.63 on the *Ca*_i_ – *Ca*_0_ phase diagram, *Ca*_*i*_ and *Ca*_0_ being the capillary numbers of inner and outer fluids, respectively. The mode is featured with a periodic, constant-speed thinning of the inner liquid tip and periodic formation of a sequence of droplets. The droplet number *n* in a sequence is determined by and increases with outer phase capillary number, and varies from two to over ten. The distribution of both pinch-off time and size of the droplets in a sequence is a geometric progression of common ratio that depends exclusively on and increases monotonically with the droplet number from its minimum value of 0.5 at *n* = 2 to its maximum value of 1 as *n* tends to infinity. These features can help identify the unique geometric morphology of droplet clusters and make them promising candidates for encryption and anti-fake identification.

Emulsions are useful in a wide range of applications, particularly when the droplet size is fine and uniform^1^,^2^,^3^. Many personal care products, foods and pharmaceutical products are emulsion-based, and emulsions have been proposed for arterial injection chemotherapy of hepatocellular carcinoma^4^, for single-molecule polymerase chain reaction (PCR)^5^, for screening of biological and synthetic compounds^6^ and for decontamination of surfaces infected by bacteria or bioterror agents^7^. Emulsification methods are plentiful^1^,^8^, but most involve mixing two liquids in bulk processes, and many use turbulence to enhance the formation of dispersions. In these “top-down” approaches , little control over the formation of individual droplets is available, and a broad distribution of size is typically produced^9^.

In an attempt to produce monodisperse emulsions, “bottom-up” microfluidic approaches— microchannel-terrace^10^,^11^,^12^,^13^, co-flow^14^, flow-focusing^15^,^16^,^17^, T-junction^18^,^19^,^20^,^21^,^22^, and capillary devices^23^,^24^,^25^, —have been recently proposed for fabricating emulsions at the level of individual droplets. All these microfluidic approaches involve the injection of one immiscible fluid into another, and operate in the laminar flow region and generate one droplet at a time. As the conditions for break-off are identical for every droplet, the emulsions fabricated by these approaches are highly monodisperse, with standard derivations in droplet size less than 5% for microchannel-terrace approach and even 2% for the others.

Introduction of one immiscible fluid into another normally leads to the formation of either droplets or a continuous jet. This comes from the Rayleigh-Plateau instability, the inner or dispersed fluid becoming unstable because of surface tension force seeking to minimize the interfacial area according to the thermodynamic principle of minimum interfacial energy. Viscous and inertial forces counteract to this action via suppressing the growth of jet deformations that lead to pinch-off and promoting the formation of a long fluid thread, respectively. It is the balance of these forces that determines droplet breakup modes for a given set of conditions arising from flow rates of the two immiscible fluids, their viscosities, densities and interfacial tension, surface wettability and device geometry. In a capillary microfluidic device (Fig. 1(a)), three modes have previously been observed^25^: geometry-controlled mode where the dimensions of the focusing orifice control the pinch-off of droplets, dripping mode with which the inner fluid finger narrows because of viscous shear force from the outer fluid, and the resulting droplet sizes are somewhat smaller than the orifice dimension, and jetting mode where a long viscous jet forms and finally breaks into droplets due to Rayleigh-Plateau instability. However, fundamental questions like whether other modes exist and how they progress as governing parameters vary remain largely unaddressed.

We report a new mode named “tip-multi-breaking” (TMB) in which the dispersed liquid tip breaks up repeatedly into several droplets once a time, and tipstreaming mode that have been observed in the other microfluidic systems on top of the three reported geometry-controlled, dripping and jetting modes in the capillary microfluidic device, as shown in Fig. 1(a). Although droplets generated with this TMB mode are polydisperse, the sizes of droplets in a cluster obey a regular distribution of geometric progression. Even more remarkably, the number of droplets in a cluster has a specific relationship to the common ratio of the progression. These features can help identify the unique geometric morphology of droplet clusters and make them promising candidates for encryption and anti-fake identification.

We also examine the transition between modes via systematically varying the dispersed and continuous phase capillary numbers that characterize the balance between local viscous shear forces and capillary pressure. The relative dominance of fluid inertia to capillary pressure is characterized by the dimensionless Weber number. At the micrometer length scale, however, fluid inertia is often negligible compared with interfacial and viscous effects.

## Experiments and Results

**Figure 1:**
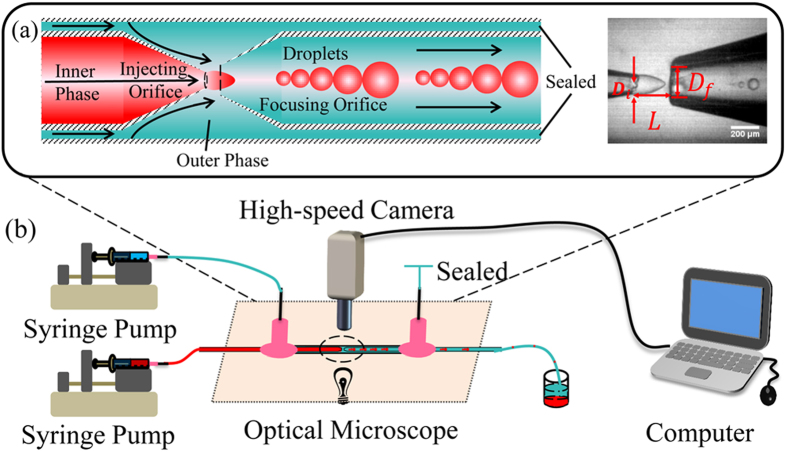
Experimental setup. (**a**) The location of injecting and focusing orifice. *D*_*i*_ and *D*_*f*_ are the inner diameters of the injecting and focusing orifice, respectively, with the distance between them being *L*. Scale bar, 200 *μm*. (**b**) Experimental setup. Fluids are driven by Syringe pumps. The right hand side needle is open before experiments, through which air and liquid wastes can be flushed away, while it is sealed during experiments. The process is visualized, monitored and recorded (in terms of images and videos) with a high speed digital imaging system equipped with an inverted microscope.

In our experiments, water-in-oil emulsion is synthesized in a capillary microfluidic device made by co-axially aligning of two cylindrical glass capillaries in a square glass capillary (Fig. 1(a)), similar to the one used by Utada *et al.*^23^ Cylindrical capillary is tapered to form injecting and focusing orifices. The system geometrical dimensions are: *D*_*f*_ = 199.15 *μm*, *L* = 266.67 *μm*. Figure 1(b) shows the system and the process. The continuous phase is silicone oil (viscosity, 881.02 mPa s); the dispersed phase is an aqueous mixture (viscosity, 19.07 mPa s) of 70 wt.% glycerol and 30 wt.% distilled water. The interfacial tension between two phases is γ = 30.07 mN m^−1^. The viscosity and interfacial tension are measured by a viscometer (microVISCTM, RHEOSENSE, INC.) and a ring tensiometer (Surface Tensiometer 20, Cole-Parmer), respectively. Using high-precision syringe pumps, these two fluids are forced to flow through the capillary microfluidic system. Flow rates are controlled and measured by the syringe pumps. The flow focusing in the microfluidic system accurately generates droplets at the focusing orifice of the output channel of the capillary microfluidic system, which allows droplets to vent to the collecting container at atmosphere pressure with the continuous fluid. All connections between the syringes and the microfluidic system and between the microfluidic system and the collecting container are via polyethylene microtubing. The droplet breakup process is visualized, monitored and recorded (in terms of images and videos) with a high speed digital imaging system equipped with an inverted microscope [high-speed camera (MotionPro® X4, IDT, Taiwan) mounted on a long distance microscope (XD101, Nanjing Jiangnan Novel Optics Co. Ltd) via a C-mount coupler].

**Figure 2:**
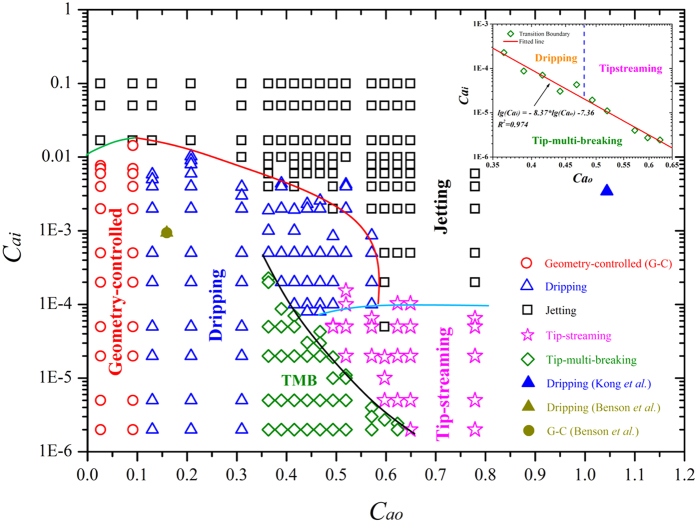
*Ca*_i_–*Ca*_o_ phase diagram. Square represents jetting, circle geometry-controlled, triangle dripping, star tipstreaming, and diamond tip-multi-breaking. Results in non-filled symbols are from our experiments; those in filled ones are from literature^24^,^25^. The variation of the present results from those in the literature^24^,^25^ shows the significant influence of device geometry. Solid lines are boundaries of transitions between different modes. Inset: transition boundaries among dripping, jetting and TMB in a log-log plane.

**Figure 3:**
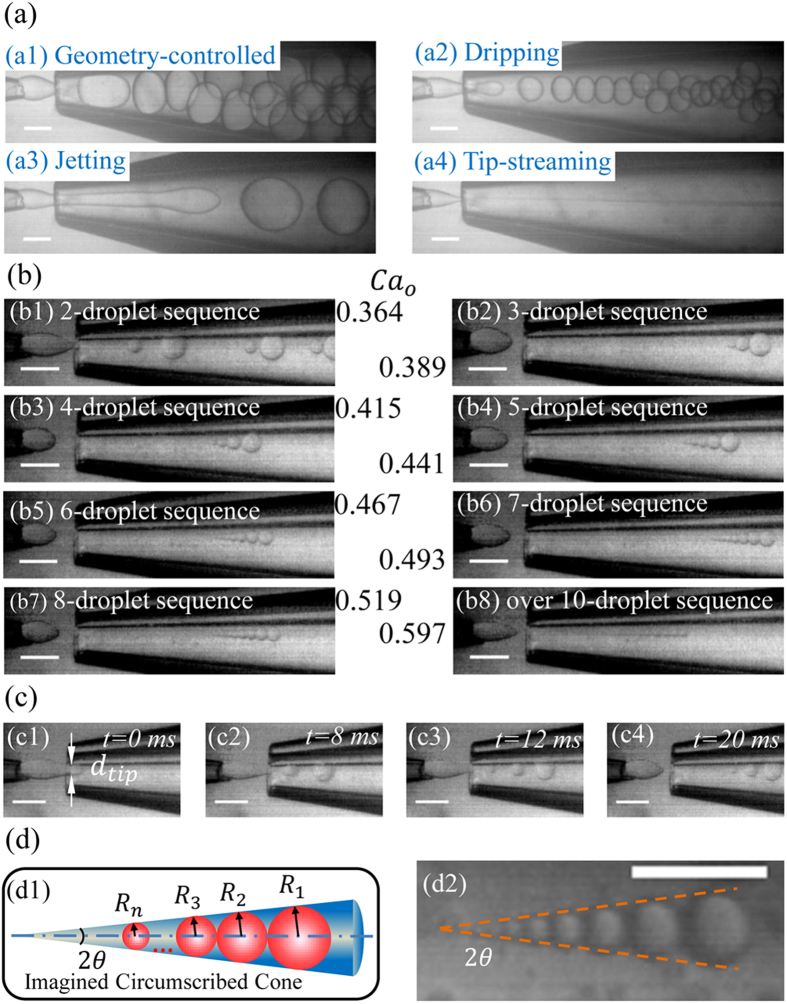
Five modes of droplet breakup. (**a**) Snapshots of four already known modes. Geometry-controlled (a1) at *Ca*_*o*_ = 0.026 and *Ca*_*i*_ = 0.0078, dripping (a2) at *Ca*_*o*_ = 0.26 and *Ca*_*i*_ = 0.0039, jetting (a3) at *Ca*_*o*_ = 0.52 and *Ca*_*i*_ = 0.0113, and tipstreaming (a4) at *Ca*_*o*_ = 0.62 and *Ca*_*i*_ = 9.8 × 10^−5^. (**b**) Tip-multi-breaking mode generates drop sequences with different droplet number that increases with outer phase capillary number *Ca*_*o*_ with *Ca*_*i*_ = 2.3 × 10^−4^, 9.1 × 10^−5^, 7.2 × 10^−5^, 4.3 × 10^−5^, 3.1 × 10^−5^, 1.9 × 10^−5^, 1.1 × 10^−5^, 2.5 × 10^−6^ for (b1-b8), respectively. (**c**) Periodic breakup process exampled by the formation of a 3-droplet sequence. (c1) Inner tip penetrates into focusing orifice, (c2-c3) thinning-tip multi-breaks into three droplets, and (c4) inner liquid tip retracts. *d*_*tip*_ denotes the diameter of the tip at the focusing orifice. (**d**) Schematic (d1) and snapshot (d2) of a circumscribed cone of apex angle 2*θ* that encloses all the droplets tangentially when they are lined adjacently in a descending order. Scale bars, 200 *μm*.

We calculate outer and inner phase capillary numbers as 
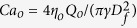
, and 
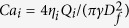
, with *η*, *Q*, *D*_*f*_, and *γ* being dynamic viscosity, volume flow rate, inner diameter of the focusing orifice, and interfacial tension, respectively. Subscripts “*o*” and “*i*” represent outer and inner phases, respectively. By systematically varying *Ca*_*i*_ and *Ca*_*o*_, we observe five modes of droplet breakup: geometry-controlled, dripping, jetting, tipstreaming and tip-multi-breaking, Fig. 2. Of them, the dripping mode has been observed at *Ca*_*o*_ = 1.044, *Ca*_*i*_ = 3.43 × 10^−3,^^24^ and *Ca*_*o*_ = 0.159, *Ca*_*i*_ = 9.4 × 10^−4,^^25^, respectively (Fig. 2). At *Ca*_*o*_ = 0.159, *Ca*_*i*_ = 9.4 × 10^−4^, but with a larger *L* value, the droplet breakup has been reported to transfer into the geometry-controlled mode^25^, showing the significance of system geometry. The jetting mode was also observed by Benson *et al.* when the surfactant is added to the outer fluids^25^. To our best knowledge, this is the first report of the tipstreaming mode in capillary device, which was previously observed in PDMS-based flow-focusing microfluidic systems^15^,^16^. Fig. 3(a) typifies these four modes from our experiments (Supplementary Movie S1). Droplets generated with geometry-controlled, dripping and tipstreaming mode are uniform, but polydisperse with jetting. With tipstreaming mode, submicron droplets can be generated at the pointed end of inner liquid tip even using a geometrical orifice of a few hundred microns in flow-focusing system^15^,^16^. The readers are referred to literature^16^,^26^,^27^ for the excellent discussion on features of these four modes known in microfluidic droplet systems and contrasts among them.

Tip-multi-breaking is a periodic mode and generates a sequence of droplets one by one in each period. The droplet number *n* in a sequence varies from two to over ten depending on and increasing with the outer phase capillary number *Ca*_*o*_ (Fig. 3(b)). The size of droplets in a sequence, beautifully, decreases monotonically in a geometric progression with common ratio smaller than unity. The details of droplet formation with this new mode are available in Supplementary Movie S2. The liquid-liquid interface upstream of the focusing orifice penetrates into the orifice due to the pressure drop along the longitudinal axis of the device. The tip proceeds through the orifice and grows into a thread of more or less mushroom shape. The growing thread displaces and pushes away the outer liquid in the outlet channel, thereby increasing the viscous shear force from the outer liquid and elongating the thread. The thread is thermodynamically unstable due to the high surface energy of this configuration, and breaks into a sequence of *n* droplets one by one with their size decreasing in a geometric progression. During the period of *n* droplets pinch-off, the inner liquid tip stays in the orifice and keeps thinning radially in diameter until it breaks and releases the last droplet, which results in the descending size distribution of the *n* droplets. After the last breakup, the tip retracts upstream from the focusing orifice and the process is repeated. The whole droplet breakup process is exampled by a 3-droplet sequence in Fig. 3(c).

**Figure 4:**
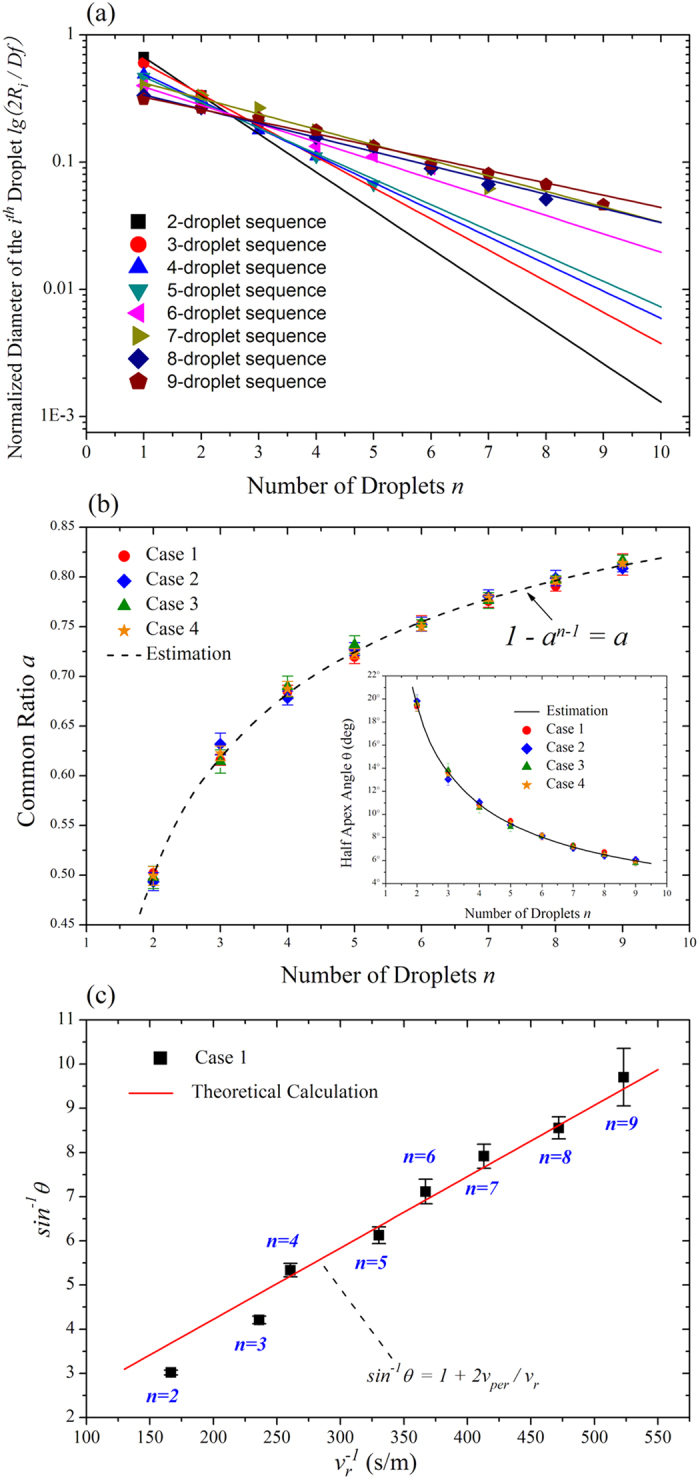
Experimental confirmation of equations (2), (4) and (7). (**a**) Experimental verification of droplet sizes obeying the rule of geometric progression. The slope of the fitted line increases with the number of droplets, indicating the common ratio of multi-droplet sequence increases with droplet number. (**b**) Common ratio *a* versus droplet number *n*. Red circle represents Case 1, blue diamond Case 2, olive triangle Case 3, and orange star Case 4 (Supplementary Table S2). The first three cases are W/O emulsions, the last one is O/W emulsion. Inset: angle *θ* versus droplet number *n*. (**c**) *sin*^−1^*θ* as a linear function of 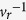 with data from Case 1.

As inertial forces are negligible compared with viscous forces at micro-length scale, fluid flow is Stokes flow in microfluidic devices. Both simulations and experiments have confirmed that drop pinch-off is featured with a constant velocity of neck radius thinning in Stokes flow regime^28^,^29^. Therefore, both the tip and the thread neck radius could be assumed to be a linear function of time so that the pinching time *t*_*i*_ of the *i*^*th*^ droplet in the sequence can be proved to obey a geometric progression (see “Confirmation of *R*_*i*_ as geometric progression” in supplementary information for the detailed proof):
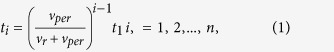
subjected to two more assumptions: the zero tip radius after the last droplet pinches off, and uniform fluid velocity *v*_*z*_ over the cross plane of the tip radius. In equation (1), *v*_*r*_ = |*dR*_*tip*_/*dt*| is the speed of tip radius thinning where *R*_*tip*_ is tip radius (Fig. 3(c1)), and *v*_*per*_ is the pinch-off velocity perpendicular to the interface leading to thread breakup due to Rayleigh-Plateau instability^23^,^28^,^29^, which depends on fluid properties in the form of 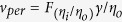
30,31. Here 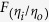 depends exclusively on the viscosity ratio *η*_*i*_/*η*_*o*_^30^,^31^. According to mass conservation, the volume of *i*^*th*^ droplet is equal to the integral of inner flow rate through the cross section of the thinning tip over the pinching time *t*_*i*_, so 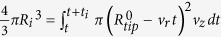, where 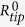 is the initial radius of the tip before the pinch-off of the first droplet. Thereby *R*_*i*_ ∝ *t*_*i*_, and *R*_*i*_ is in the form of,
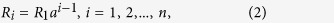
in which *R*_*i*_ is the radius of *i*^*th*^ droplet, and
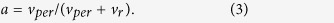
The predicted *R*_*i*_ from equation (2) is compared with experimental data in Fig. 4(a), confirming the accuracy of equation (2). Here we measure *R*_*i*_ and count *n* by analyzing the captured images with *ImageJ*. The slope of the fitted line in Fig. 4(a) is proportional to 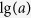. The more droplets assemble in one sequence, the larger value the slope becomes. Hence the common ratio *a* increases with droplet number *n*.

The change of tip radius up to time instant *t*_*p*_ during the pinch-off of the *n* droplets is given by the product of breakup velocity and time *t*_*p*_. By assuming the tip radius becomes zero after the formation of the last droplet, we can estimate the correlation between common ratio *a* and the droplet number *n* as follows (see “Determining the correlation between *θ* and *n*” in supplementary information):
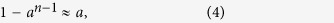
which is independent of parameters arising from fluid properties and device geometry. We confirm this in Fig. 4(b) by measuring *a* and *n* with different liquids and device dimensions listed in Supplementary Table S2, showing an excellent agreement between equation (4) and experimental data. Therefore the common ratio *a* is fixed by the droplet number exclusively. Based on equation (4), the minimum value of *a* is 0.5 and occurs at *n* = 2. Therefore, the radius of any droplet in the sequence would never be larger than the twice of that of its smaller neighbor or smaller than the half of that of its bigger neighbor. Multi-droplet sequences with different droplet number are similar to each other in the geometric morphology, but can be distinguished exclusively by the correlation between *n* and *a*. When such droplet sequences are encapsulated into shell droplets or fibers, their number and size distribution can serve as the marker of the encoded shell droplets or fibers, a unique identifier different from those proposed in the literature for encryption and anti-fake identification^32^,^33^.

As geometric progression of both pinch-off time and droplet size in any droplet sequence comes from the constant speed of tip radius thinning and has been experimentally confirmed (Fig. 4(a)), we can thus introduce a circumscribed cone with apex angle 2*θ* to embrace all the droplets in the sequence (Fig. 3(d)). A straightforward derivation yields,
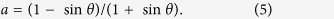
By equations (4) and (5), we have
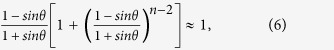
which shows that sin*θ* *≤* 1/3 when *n* ≥ 2. Therefore 0° < θ ≤ arcsin(1/3) ≈ 20° with tip-multi-breaking mode. Equation (6) is confirmed in the inset of Fig. 4(b).

By equations (3) and (5), we have
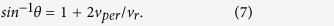
When the pinch-off velocity *v*_*per*_ = 0, the effect of Rayleigh-Plateau instability on the thread breakup vanishes, and the droplet is generated by the tip breakup one by one. This corresponds to dripping with *sinθ* = 1 and *θ* = 90°. When *v*_*r*_ = 0, *R*_*tip*_ is constant. We thus recover the jetting mode with *sinθ* = 0 and *θ* = 0° if *v*_*per*_ ≠ 0. When neither *v*_*r*_ nor *v*_*per*_ is zero, we have the tip-multi-breaking mode with 0° < *θ* < 90°. Therefore the *θ*-value can be used to distinguish dripping, jetting and tip-multi-breaking. Note that the difference between the last two is mainly on that *R*_*tip*_ keeps constant in jetting, but thinning with a constant speed in tip-multi-breaking.

Measuring 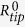 and *θ* by analyzing the captured images with *ImageJ* and calculating the total breakup time *t*_*p*_ via the captured videos, we can calculate 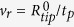. Figure 4(c) shows the experimental results of *sin*^−1^*θ* as a linear function of 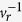, in which all the measured values of *sin*^−1^*θ* lie closely to the prediction from equation (7) with the droplet number from two to nine, showing a good agreement.

The location of the tip-multi-breaking mode and the transitions between the modes are shown in the *Ca*_*o*_–*Ca*_*i*_ phase diagram (Fig. 2). Tip-multi-breaking appears in a region formed and enveloped by a curve 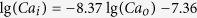 (inset in Fig. 2) as an upper boundary, and three straight lines *Ca*_*i*_ = 0, *Ca*_*o*_ = 0.35, and *Ca*_*o*_ = 0.63 as the lower, left and right boundary, respectively. In experiments, we observe transitions between modes by decreasing *Ca*_*i*_ for any fixed value of *Ca*_*o*_. The mode transfers into another in different orders for different outer phase capillary numbers. As *Ca*_*i*_ decreases, the breakup mode shifts from jetting to geometry-controlled when *Ca*_*o*_ < 0.1, from jetting to dripping when 0.1 < *Ca*_*o*_ < 0.35, from jetting to dripping and then tip-multi-breaking when 0.35 < *Ca*_*o*_ < 0.5, from jetting to dripping, tipstreaming and tip-multi-breaking when0.5 < *Ca*_*o*_ < 0.6, and from jetting to tipstreaming and then tip-multi-breaking when 0.6 < *Ca*_*o*_ < 0.63. As *Ca*_*o*_ arrives at 0.5, transition from jetting to tipstreaming is observed at *Ca*_*i*_ about 2 × 10^−4^, indicating a sufficiently low inner flow rate. Although tipstreaming reported previously are mainly surfactant mediated^15^,^16^, both experimental and numerical studies suggested that it can also occur in the absence of surfactant^34^,^35^,^36^, so does in our experiments.

## Concluding Remarks

To summarize, the pressure drop along the longitudinal axis of the device forces the liquid-liquid interface into the focusing orifice. Depending on values of *Ca*_*o*_ and *Ca*_*i*_, the tip breaks up into droplets in five modes, with their appearance domain and transition shown in Fig. 2: geometry-controlled, dripping, jetting, tipstreaming and tip-multi-breaking. The last is observed for the first time, and is characterized by a periodic, constant-speed thinning of inner tip radius, and thus generates periodically a sequence of *n* droplets (*n* ≥ 2) with their pinch-off times and sizes satisfying a geometric progression of common ratio *a*. The droplet number *n* in a sequence is determined by and increases with outer phase capillary number *Ca*_*o*_. The common ratio *a* depends exclusively on and increases monotonically with *n* from its minimum value of 0.5 at *n* = 2 to its maximum values of 1 as *n* tends to infinity. While this new droplet breakup mode is observed in capillary microfluidic devices at present work, it is believed to be a basic mode that could appear in other microfluidic systems such as flow-focusing systems as well.

## Additional Information

**How to cite this article**: Zhu, P. *et al.* Tip-multi-breaking in Capillary Microfluidic Devices. *Sci. Rep.* **5**, 11102; doi: 10.1038/srep11102 (2015).

## Supplementary Material

Supplementary Information

Supplementary Information

Supplementary Information
