# Supplementary material for: Tip-multi-breaking in Capillary Microfluidic Devices
Source: Sci Rep. 2015 Jun 16;5:11102. doi: 10.1038/srep11102 (PMC4468424; doi:10.1038/srep11102)
Supplement: Supplementary Information [file srep11102-s1.pdf]

Supplementary Information

"Tip-multi-breaking in Capillary Microfluidic Devices"

Pingan Zhu[1, 2], Tiantian Kong[1, 2], Zhanxiao Kang[1, 2], Xiaowei Tian[1, 2], Liqiu Wang[1, 2, *]

[1]Department of Mechanical Engineering, the University of Hong Kong, Hong Kong

[2]HKU-Zhejiang Institute of Research and Innovation (HKU-ZIRI), 311100, Hangzhou, Zhejiang, China

* Corresponding author: lqwang@hku.hk

This file includes:

Legends of Supplementary Movies S1 to S2

Supplementary Information: Confirmation of R_i as geometric progression

Supplementary Information: Determining the correlation between *a* and *n*

Supplementary Table S2

Supplementary Movie S1: four known breakup modes

This video demonstrates four known modes: geometry-controlled, dripping, jetting, and tipstreaming in capillary microfluidic devices. The inner phase is 70 wt.% glycerol in distilled water, and outer phase is silicone oil. The video is recorded with 1000 fps and played with 50 fps.

Supplementary Movie S2: tip-multi-breaking mode

This video shows the multi-droplet sequences with different droplet numbers from two to over ten. The inner phase is 70 wt.% glycerol in distilled water, and outer phase is silicone oil. The video is recorded with 1000 fps and played with 20 fps.

Confirmation of R_i as geometric progression

Based on the experimental observations, the series droplet sizes are presumed to obey the rule of geometric progression. An imagined circumscribed cone is introduced to enclose all the droplets when they are lined adjacently in a decreasing order, see Fig. S1(a). Then the relation between half apex angle of the cone θ and radius of the ith droplet R_i gives,

$$R_i = R_1 a^{i-1}, \quad i = 1, 2, \ldots, n, \tag{1}$$

where $a = (1 - sin\theta)/(1 + sin\theta)$ is the common ratio of the geometric progression. However, the validation of geometric progression still needs to be verified quantitatively. Kinetic analysis is employed to quantify the process of droplet sequence formation.

Several parameters are defined to illustrate the problem, see TABLE S1. Among them, t_{pi} represents the time remained after the ith droplet is generated. Because droplet is generated one after another temporally during the tip radius thinning, the relationship between t_p and t_i gives $t_p = \sum_{i=1}^{n} t_i$, and $t_{p_i} = t_p - \sum_{k=1}^{i} t_k$. v_{per} denotes the pinch-off velocity perpendicular to the liquid-liquid interface, $v_{per} = F_{(\eta_i/\eta_o)} \gamma / \eta_o$, where $F_{(\eta_i/\eta_o)}$ is a function of viscosity ratio η_i/η_o [1-3].

TABLE S1. Parameters defined in this file

t_i	time interval between the ith and $(i-1)^{th}$ droplet pinch-off
t_p	total breakup-time for a series of droplets, $t_p = \sum_{i=1}^{n} t_i$
t_{pi}	$t_{p_i} = t_p - \sum_{k=1}^{i} t_k$, time remained after the ith droplet is formed
v_z	fluid velocity along the flowing direction (Fig. S1(b))
v_r	speed of the tip radius thinning, $v_r = \lvert dR_{tip} / dt \rvert$
v_{per}	perturbation growth velocity perpendicular to the interface
R_{tip}^{i} [a]	tip radius after the ith droplet is formed (Fig. S1(c), (d))
D_f	inner diameter of focusing orifice (Fig. S1(c))
R_{neck}^{i}	neck radius at the beginning of the ith droplet pinch-off (Fig. S1(d))

[a] R_{tip}^{0} is the initial radius of the tip when no droplet is formed.

According to literature[4,5], both the inner liquid tip and thread neck radii (Fig. S1(d)) are assumed to be thinning constantly with time during all the breakup process, and after all the droplets are generated, $R_{tip} = 0$, so that R_{tip}^{i} is given as

$$R_{tip}^{i} = -v_r t = v_r t_{pi}, \tag{2}$$

as illustrated in Fig. S1(e).

We also assume that at the start of the ith droplet pinch-off, the mushroom-shaped neck radius R_{neck}^{i} (Fig. S1(d)) is equal to the tip radius R_{tip}^{i-1}, and the breakup is controlled by the combined effect of v_r and v_{per}, so for the ith droplet breakup during time interval t_i, an estimation is achieved (see Fig. S1(e)),

$$(v_r + v_{per}) t_i \approx R_{neck}^{i} = R_{tip}^{i-1}. \tag{3}$$

Solving Eqs. (2) and (3) leads to,

$$t_{p_{i-1}} = (v_r + v_{per}) t_i / v_r. \tag{4}$$

Substituting $t_{pi} = t_{pi-1} - t_i$ into Eq. (4), we finally have

$$t_{p_i} = \frac{v_{per}}{v_r + v_{per}} t_{pi-1}, \tag{5a}$$

hence,

$$t_{p_i} = \left(\frac{v_{per}}{v_r + v_{per}} \right)^i t_p, \tag{5b}$$

such that, based on Eqs. (4) and (5a),

$$t_{i+1} = \frac{v_{per}}{v_r + v_{per}} t_i, \quad i = 1, 2, \ldots, n. \tag{6}$$

Eq. (6) states that t_i is a geometric progression, for both v_r and v_{per} are constant for a fixed system.

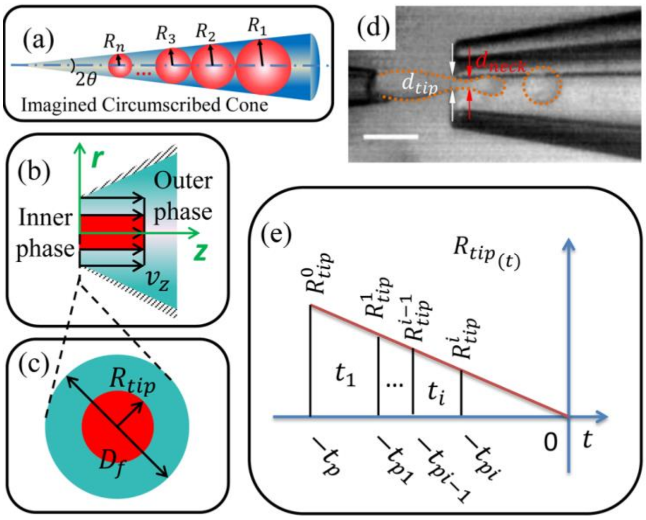

Figure S1| Schematic of the model. (a) Imagined circumscribed cone covering all the droplets tangentially. The apex angle is 2θ. (b) Flat velocity profile of v_z at the entrance of focusing orifice. (c) Cross-section plane at the entrance of the focusing orifice. R_{tip} is the radius of the tip, and D_f denotes the inner diameter of the focusing orifice. (d) Snapshot shows the tip diameter d_{tip} and thread neck diameter d_{neck}. At the beginning of every single droplet breakup, they are assumed to have the same value, and then the neck radius ($d_{neck}/2$) diminishes to zero with velocity v_{per} while tip radius ($d_{tip}/2$) thinning with velocity v_r. Scale bar is 200 μm. (e) Schematic of the linear relationship between inner tip radius R_{tip} and time.

At the entrance of the focusing orifice, v_z could be assumed to be with flat velocity profile, as shown in Fig. S1(b), and the volume of the ith droplet is calculated by the integral of the flow rate through the cross-section of the thinning tip over time (see Figs. S1(b) and (e)), so that

$$\frac{4}{3}\pi R_i^3 = \int_{-t_{p_{i-1}}}^{-t_{p_i}} \pi\left(-v_r t\right)^2 v_z dt = \frac{\pi}{3} v_z v_r^{\,2}\left(t_{p_{i-1}}^{\ 3} - t_{p_i}^{\ 3}\right), \tag{7}$$

Replacing t_{pi-1} and t_{pi} by t_i, Eq. (7) gives,

$$\frac{4}{3}\pi R_i^3 = \frac{\pi}{3} v_z v_r^{\,2}\left[\left(\frac{v_r + v_{per}}{v_r}\right)^3 - \left(\frac{v_{per}}{v_r}\right)^3\right] t_i^{\,3}, \tag{8}$$

As v_r, v_z and v_{per} are all constant, Eq. (8) shows $R_i \propto t_i$, which confirms the presumption of R_i as a geometric progression with common ratio $a = v_{per}/(v_r + v_{per})$.

Determining the correlation between *a* and *n*

If $v_{per} = m v_r$, where *m* is a constant ratio, we thus have,

$$\left(v_{per} + v_r\right) t_p = (m+1) v_r t_p = (m+1) R_{tip}^0 . \tag{9a}$$

The term at left hand side in Eq. (9a) represents the total length change of the thread height during the whole pinch-off time t_p, which is the sum of the *n* thread neck heights $\sum_{i=1}^{n} R_{neck}^i$ and the variation of the tip radius R_{tip}^0. However, R_{neck}^n is double-counted since R_{tip} and R_{neck} coincide with each other and diminish together to zero during the last droplet breakup. As mentioned above, $R_{neck}^i = R_{tip}^{i-1}$, so

$$\left(v_{per} + v_r\right) t_p = \sum_{i=1}^{n} R_{neck}^i + R_{tip}^0 - R_{neck}^n = \sum_{i=1}^{n-1} R_{neck}^i + R_{tip}^0 = \sum_{i=1}^{n-1} R_{tip}^{i-1} + R_{tip}^0 . \tag{9b}$$

Combining Eqs. (2), (5b), (9a), and (9b) yields,

$$1 - a^{n-1} \approx a, \tag{10}$$

where $a = v_{per}/(v_r + v_{per}) = m/(m+1)$ is the common ratio. Eq. (10) correlates the common ratio *a* and the number of droplets *n*, which is independent of any other parameters such as material property and device geometry.

Note that all the derivations above are based on three assumptions: 1) linear thinning of both inner liquid tip and thread neck radii; 2) the zero tip radius after the last droplet pinches off; and 3) uniform fluid velocity v_z over the cross plane of the tip radius. These assumptions hold until the singularity occurs at the final stage of droplet pinch-off[6,7].

Supplementary Table S2: Material property and device geometry

Four Cases			η (mPa s)	γ (mN m^{-1})	D_f (μm)
Case 1	Inner Phase	70 wt.% glycerol	19.07	30.07	199.15
	Outer Phase	Silicone Oil	881.02		
Case 2	Inner Phase	70 wt.% glycerol	19.07	21.35	190.68
	Outer Phase	Silicone Oil + 1wt% DC749	854.46		
Case 3	Inner Phase	70 wt.% glycerol	19.07	21.75	173.73
	Outer Phase	Soybean Oil	93.17		
Case 4	Inner Phase	Silicone Oil	95.75	29.43	165.59
	Outer Phase	95 wt.% glycerol	327.43		

References

1 Utada, A. *et al.* Monodisperse double emulsions generated from a microcapillary device. *Science* **308**, 537-541 (2005).

2 Powers, T. R., Zhang, D., Goldstein, R. E. & Stone, H. A. Propagation of a topological transition: The Rayleigh instability. *Phys. Fluids* **10**, 1052-1057 (1998).

3 Powers, T. R. & Goldstein, R. E. Pearling and Pinching: Propagation of Rayleigh Instabilities. *Phys. Rev. Lett.* **78**, 2555-2558 (1997).

4 Zhang, W. W. & Lister, J. R. Similarity solutions for capillary pinch-off in fluids of differing viscosity. *Phys. Rev. Lett.* **83**, 1151 (1999).

5 Cohen, I. & Nagel, S. R. Testing for scaling behavior dependence on geometrical and fluid parameters in the two fluid drop snap-off problem. *Phys. Fluids* **13**, 3533-3541 (2001).

6 Eggers, J. & Villermaux, E. Physics of liquid jets. *Rep. Prog. Phys.* **71**, 036601 (2008).

7 Doshi, P. *et al.* Persistence of memory in drop breakup: The breakdown of universality. *Science* **302**, 1185-1188 (2003).
